# Ileal intubation is not associated with higher detection rate of right-sided conventional adenomas and serrated polyps compared to cecal intubation after adjustment for overall adenoma detection rate

**DOI:** 10.1186/s12876-019-1111-0

**Published:** 2019-11-15

**Authors:** Martin Buerger, Philipp Kasper, Gabriel Allo, Johannes Gillessen, Christoph Schramm

**Affiliations:** Department for Gastroenterology and Hepatology, Faculty of Medicine and University Hospital Cologne, Kerpener Strasse 62, 50937 Cologne, Germany

**Keywords:** Colorectal cancer, Endoscopy, Colonoscopy, Screening, Serrated polyp, Adenoma, Detection rate, Cecal intubation, Ileal intubation

## Abstract

**Background:**

High cecal intubation rate (CIR) is associated with significant improved adenoma detection rate (ADR), however, self-reported CIR may be overestimated and inadequate documentation of cecal intubation is associated with a lower polyp detection rate compared to clear documentation. We aimed to investigate if ileal intubation may be associated with higher detection rates (DR) for right-sided conventional adenomas (cAD) and serrated polyps (SP) compared to cecal intubation in a large screening colonoscopy cohort.

**Material and methods:**

Retrospective analysis of individuals ≥50 years with average risk for colorectal cancer (CRC) who underwent screening colonoscopy between 01/01/2012 and 14/12/2016 at a tertiary academic hospital and six community-based private practices. Exclusion criteria were conditions with increased risk for CRC (e.g. inflammatory bowel disease, history of CRC, hereditary cancer syndromes), previous colonoscopy at the same institution, and incomplete procedures. Right-sided colon was defined as caecum and ascending colon.

**Results:**

4.138 individuals were analysed (mean age 62 years, 52.1% female). DR for right-sided cADs and SPs were significantly higher after ileal compared to cecal intubation in univariate (12.5% vs. 6.8%, *p* < 0.001, and 6.3% vs. 3.3%, *p* < 0.001), but not in multivariate analysis (OR 1.025, 95%-CI 0.639–1.646, *p* = 0.918, and OR 0.937, 95%-CI 0.671–1.309, *p* = 0.704). DRs did not differ between ileal and cecal intubation for endoscopists with ADR ≥25 and < 25%, respectively. ADR ≥25% was significantly associated with ileal intubation (OR 21.862, 95%-CI 18.049–26.481, *p* < 0.001).

**Conclusion:**

Ileal intubation may not provide any benefit over cecal intubation concerning the detection of cADs and SPs in the right-sided colon.

## Background

Colorectal cancer (CRC) is one of the most frequent cancers worldwide with an estimated age-standardized incidence rate of 23.1/100.000 and with 1.8 million newly diagnosed cases worldwide in 2018 [1]. It develops through the adenoma-carcinoma-sequence and the serrated pathway which accounts for up to one third of all CRCs [2]. Serrated polyps (SPs) which comprise hyperplastic polyps (HPs), sessile serrated adenomas (SSAs) and traditional serrated adenomas (TSAs) according to the WHO classification represent the precursor lesions of the serrated pathway [3]. Because CRC is highly suitable for screening procedures, CRC screening programs have been implemented in many countries over the last two decades [4]. Screening colonoscopy was shown to be associated with a significant reduction in CRC-incidence and mortality of 66 and 69%, respectively [4]. However, the efficacy of screening colonoscopy is less pronounced in the proximal colon [5–9]. These findings are ascribed to interval cancers which are defined as the occurrence of CRC after screening colonoscopy and before the next scheduled colonoscopy and which occur in up to 3.7% (95%-CI 2.8–4.9%) of screened individuals [10]. Interval cancers are mainly attributed to missed or incompletely resected lesions and are associated with a proximal localization and a serrated histology [11–13]. A meta-analysis of six studies comprising 465 patients demonstrated a pooled adenoma miss rate of 22% (95%-CI 19–26) [14].

The completeness of the examination of the colon during screening colonoscopy is assured by the intubation of the cecum. Cecal intubation rate (CIR) is defined as the percentage of procedures reaching and visualizing the whole cecum and its landmarks [15]. It should be documented in a written report as well as with photo or video documentation [15]. The European Society of Gastrointestinal Endoscopy recommends a minimum CIR of ≥90% and a target CIR of ≥95% [15]. Large studies demonstrated a significant association between CIR and adenoma detection rate (ADR) in the entire, proximal and right-sided colon [16, 17]. Lower CIRs are associated with a higher risk for interval cancer in a proximal as well as distal localization [18]. A recent study revealed significant differences between self reported and audited CIR [19]. Furthermore, lower polyp detection rates were observed in colonoscopies with an inadequate documentation of cecal intubation compared to colonoscopies with a clear documentation in another recent study [20]. We, therefore, hypothesised that polyp detection rates may be higher in colonoscopies with ileal intubation which definitely secures complete examination of the colon compared to procedures with self reported cecal intubation. The aim of this study was to investigate if ileal intubation may be associated with higher detection rates of right-sided conventional adenomas and SP compared to cecal intubation in a large screening colonoscopy cohort.

## Methods

Consecutive screening colonoscopies were identified on a clinical case-base from multiple prospectively operated endoscopy databases (*n* = 7), in which all endoscopic procedures performed in each participating centre have been prospectively documented, and analysed retrospectively. Inclusion criteria were age ≥ 50 years, average risk for CRC (i.e. conditions with increased risk for CRC like inflammatory bowel disease, history of CRC and hereditary cancer syndromes were not present) and complete colonoscopy (i.e. reported cecal intubation). Exclusion criteria were procedures with scheduled polypectomy and previous colonoscopy at the same institution. All procedures were performed by 15 experienced endoscopists (i.e. ≥300 colonoscopies annually for any indication) between 01/01/2012 and 14/12/2016 at a tertiary academic hospital and six community-based private practices. We assumed a similar level of experience amongst participating endoscopists because of high numbers of colonoscopies performed by each endoscopist and we, therefore, did not adjust for this factor. There was no obligation for endoscopists to intubate the terminal ileum during colonoscopy. Conventional adenomas included tubular, tubulovillous and villous adenomas. SPs included HPs, SSAs and TSAs. SPs and conventional adenomas were summarised as neoplastic polyps. Original histopathological diagnosis, reported by the participating centre, was used to classify colorectal polyps into conventional adenomas and SPs. Detection rate was defined as the percentage of procedures in which at least one polyp of a certain histological subtype was detected. The right-sided colon included the cecum and the ascending colon. The quality of bowel preparation for the entire colon was retrospectively evaluated on the basis of the endoscopy report and classified into adequate (excellent, good, fair) and poor.

Statistical analysis was performed using Statistical Package for the Social Sciences statistics version 24 (IBM, Chicago, USA) and MS Excel (Microsoft, Richmond, USA). Categorical variables were analyzed as absolute numbers and their relative frequencies, and compared using χ^2^-test. Age as continuous variable was analyzed as median and interquartile range (IQR). Logistic regression analysis was performed with ileal intubation and detection rates, respectively, as dependent variables. A *p*-value < 0.05 was considered as statistically significant.

Because of the strictly retrospective design of our study, approval by a local ethics committee and written informed consent from the participants were not required, in accordance with German law (paragraph 15, sentence 1, North Rhine Medical Association’s professional code of conduct from 14 November 1998 as amended on 19 November 2011, and paragraph 6, sentence 1, Health Data Protection Act of North Rhine-Westphalia).

## Results

A total of 4138 individuals (52.1% females) with a median age of 62.0 years (IQR 56–69) were included in the analysis. The overall ADR was 31.9%. CIR was 100% as defined by inclusion criteria. The terminal ileum was intubated in 78.1% of the procedures. Endoscopists with an ADR ≥25% (*n* = 9) had significantly higher ileal intubation rates (IIR) compared to endoscopists with an ADR < 25% (*n* = 6) (84.2% vs. 15.8%, *p* < 0.001). Also, gender (79.6% in male vs. 76.6% in female, *p* = 0.019), presence of colonic diverticula (82.0% vs. 75.6%, *p* < 0.001) and propofol sedation (78.3% vs. 55.0%, *p* < 0.001) were significantly associated with a higher IIR in univariate analysis, whereas this was not found for age (median age 62.0 vs. 62.0 years, *p* = 0.196) and quality of bowel preparation (77.4% for adequate vs. 75.7% for poor, *p* = 0.653). Using logistic regression analysis, ADR and propofol sedation remained significantly associated with IIR (Table 1).

**Table 1:** Logistic regression analysis of variables associated with ileal intubation rate

	OR	95%-CI	p
Female gender	0.865	0.717–1.043	0.129
Diverticulosis	1.091	0.897–1.328	0.382
Propofol sedation	2.233	1.611–3.095	< 0.001
ADR ≥25%	21.862	18.049–26.481	< 0.001

*OR* odds ratio, *CI* confidence interval, *ADR* adenoma detection rate

A total of 1008 polyps were detected in the right-sided colon, of which 908 (90.1%) were neoplastic (290 SP, 615 conventional adenomas, 3 cancers). Two hundred sixty-two neoplastic polyps (89 SP, 171 conventional adenomas, 2 cancers) were located within the caecum and 646 neoplastic polyps (201 SP, 444 conventional adenomas, 1 cancer) were located in the ascending colon. The relative proportion of SP did not differ between the caecum and the ascending colon (33.9% vs. 33.1%).

Detection rates for neoplastic polyps, SP and conventional adenomas in the right-sided colon were significantly higher, when the colonoscope was inserted into the terminal ileum compared to the intubation of the caecum alone (Table 2). This was also found when detection rates were separately analysed for the caecum (except SP) and the ascending colon as well as for gender (except neoplastic polyps in the caecum in female, SP in the caecum in male and female and in the ascending colon in male, and conventional adenomas in the caecum in male and female).

**Table 2:** Detection rates (DR) for neoplastic polyps, serrated polyps (SP) and conventional adenomas (cAD)

DR [%]		Overall	Ileal intubation	Cecal intubation	p
Neoplastic polyps	Right-sided colon	16.5	18.1	10.6	< 0.001
	Caecum	5.6	6.1	3.8	0.008
	Ascending colon	12.4	13.7	7.7	< 0.001
SP	Right-sided colon	5.7	6.3	3.3	< 0.001
	Caecum	1.9	2.0	1.2	0.074
	Ascending colon	4.0	4.5	2.2	0.001
cAD	Right-sided colon	11.3	12.5	6.8	< 0.001
	Caecum	3.7	4.0	2.6	0.045
	Ascending colon	8.4	9.4	4.8	< 0.001

Endoscopists with ADR ≥25% had significantly higher detection rates for all polyp entities (neoplastic polyps, SP and conventional adenomas) and all localization (right-sided colon as well as caecum and the ascending colon) than endoscopists with ADR < 25% (Table 3). However, we did not observe significant differences in detection rates as a function of maximum insertion of the colonoscope (ileal vs. cecal intubation) within the groups of endoscopists with ADR ≥25% and ADR < 25%, respectively (Table 3). Results from logistic regression analysis of detection rates for SP and conventional adenomas as dependent variables in the right-sided colon, caecum, and ascending colon are presented in Tables 4 and 5, respectively. ADR ≥25% was associated with an increased chance to detect SP and conventional adenomas in the right-sided colon as well as in the caecum and the ascending colon, whereas gender was associated only with conventional adenomas, but not SP. Ileal intubation, propofol sedation, diverticulosis, and quality of bowel preparation, however, did not influence the detection of SP and conventional adenomas in the right-sided colon.

**Table 3:** Detection rates (DR) for neoplastic polyps, serrated polyps (SP) and conventional adenomas (cAD)

DR%			Overall	Ileal intubation	Cecal intubation	p
Neoplastic polyps	Right-sided colon	ADR < 25%	7.6	7.7	7.6	0.951
		ADR ≥ 25%	20.2	20.1	21.8	0.556
	Caecum	ADR < 25%	2.4	2.6	2.3	0.679
		ADR ≥ 25%	6.9	6.7	6.7	0.114
	Ascending colon	ADR < 25%	5.4	5.1	5.6	0.676
		ADR ≥ 25%	15.3	15.3	15.7	0.867
SP	Right-sided colon	ADR < 25%	2.3	1.9	2.7	0.356
		ADR ≥ 25%	7.1	7.2	5.6	0.396
	Caecum	ADR < 25%	0.5	0.6	0.5	0.944
		ADR ≥ 25%	2.4	2.3	3.6	0.277
	Ascending colon	ADR < 25%	1.8	1.3	2.1	0.275
		ADR ≥ 25%	5.0	5.1	2.5	0.104
cAD	Right-sided colon	ADR < 25%	4.8	5.6	4.3	0.262
		ADR ≥ 25%	14.0	13.8	16.2	0.341
	Caecum	ADR < 25%	1.6	1.9	1.5	0.569
		ADR ≥ 25%	4.6	4.4	7.1	0.084
	Ascending colon	ADR < 25%	3.3	3.8	2.9	0.417
		ADR ≥ 25%	10.5	10.5	11.7	0.593

*ADR* adenoma detection rate

**Table 4:** Logistic regression analysis of detection rates for serrated polyps (SP)

		OR	95%-CI	p
SP right-sided colon	ADR ≥25%	3.771	2.294–6.199	< 0.001
	Male gender	0.830	0.631–1.091	0.182
	Ileal intubation	1.000	0.620–1.613	0.998
	Propofol sedation	0.746	0.442–1.261	0.274
	Diverticulosis	0.869	0.658–1.148	0.323
	Adequate BP	1.648	0.662–4.098	0.283
SP Caecum	ADR ≥25%	9.600	3.391–27.172	< 0.001
	Male gender	0.685	0.426–1.103	0.120
	Ileal intubation	0.557	0.266–1.168	0.121
	Propofol sedation	0.533	0.236–1.203	0.130
	Diverticulosis	1.050	0.659–1.673	0.837
	Adequate BP	2.905	0.395–21.349	0.295
SP Ascending colon	ADR ≥25%	2.792	1.605–4.857	< 0.001
	Male gender	0.906	0.658–1.248	0.546
	Ileal intubation	1.282	0.719–2.287	0.400
	Propofol sedation	0.855	0.451–1.621	0.632
	Diverticulosis	0.783	0.562–1.090	0.147
	Adequate BP	1.427	0.517–3.937	0.492

*OR* odds ratio, *CI* confidence interval, *ADR* adenoma detection rate, *BP* bowel preparation

**Table 5:** Logistic regression analysis of detection rates for conventional adenomas (cAD)

		OR	95%-CI	p
cAD right-sided colon	ADR ≥25%	3.385	2.398–4.777	< 0.001
	Male gender	1.507	1.230–1.845	< 0.001
	Ileal intubation	0.924	0.660–1.293	0.643
	Propofol sedation	0.833	0.571–1.215	0.343
	Diverticulosis	1.116	0.912–1.365	0.287
	Adequate BP	1.343	0.744–2.422	0.327
cAD Caecum	ADR ≥25%	3.497	1.959–6.243	< 0.001
	Male gender	1.644	1.171–2.307	0.004
	Ileal intubation	0.705	0.414–1.199	0.197
	Propofol sedation	0.893	0.482–1.655	0.720
	Diverticulosis	1.110	0.796–1.548	0.540
	Adequate BP	2.032	0.633–6.516	0.233
cAD Ascending colon	ADR ≥25%	3.686	2.452–5.541	< 0.001
	Male gender	1.479	1.174–1.862	0.001
	Ileal intubation	0.938	0.636–1.385	0.938
	Propofol sedation	0.859	0.557–1.324	0.490
	Diverticulosis	1.097	0.873–1.379	0.428
	Adequate BP	1.294	0.666–2.512	0.447

*OR* odds ratio, *CI* confidence interval, *ADR* adenoma detection rate, *BP* bowel preparation

## Discussion

The quality of self reported cecal intubation rate during colonoscopy has been addressed by two recent studies. The first study, a retrospective study from the United Kingdom, demonstrated that endoscopists who documented cecal intubation by a clear image had a significantly higher polyp detection rate in the entire colon (OR 2.1, 95%-CI 1.4–3.2, *p* = 0.001) as well as in the right-sided colon (OR 3.67, 95%-CI 1.91–7.02, *p* < 0.001) than endoscopists who provided no or an unclear image [20]. In the second study from Poland, the video documented cecal intubation rate was significantly lower than the self reported cecal intubation rate (84.4% vs. 96.6%, *p* = 0.001) [19].

In our study of a large cohort of individuals with an average CRC-risk who underwent screening colonoscopy, we observed significantly higher detection rates for conventional adenomas and SPs in the right-sided colon when the terminal ileum was intubated during the procedure compared to cecal intubation alone in univariate analysis. However, these findings were abolished when we analysed detection rates separately for endoscopists with high (≥25%) and low (< 25%) ADR, respectively. Multivariate analysis identified individual endoscopist’s ADR as the most important variable for the detection of right-sided conventional adenomas and SP, whereas gender was only associated with right-sided conventional ADR but not SP detection rate. We previously showed that ileal intubation was not superior to cecal intubation in terms of ADR in the entire colon [21].

The importance of detecting proximal and right-sided colonic lesions has been pointed out by several studies which demonstrated a reduced efficacy of screening colonoscopy to prevent CRC in the proximal colon [5–9]. Consequently, efforts have been made to increase proximal detection rates. Retroflexion of the colonoscope in the caecum enables the endoscopist to look behind the folds of the right-sided colon. Technical success rates of this technique > 90% were reported with loop formation being the most common reason for failed retroflexion [22–24]. Up to 16.8% additional adenomas compared to a single or dual inspection of the right-sided colon in forward view were reported with an increase of polyp detection rate and ADR from 28.57 to 30.57% and from 24.64 to 26.4%, respectively [22, 23]. A recent systematic review and meta-analysis including five studies with 4155 patients, however, demonstrated that a second inspection of the right-sided colon, irrespective of forward-view or retroflexion, leads to modest improvement in proximal detection rates [25]. Advanced endoscopic imaging modalities like narrow band imaging, linked color imaging and I-scan were linked with increased detection rates in the right-sided colon in recent studies [26–28]. Additionally, device assisted endoscopy was shown to be significantly associated with higher detection rates. Cap-assisted colonoscopy increased right-sided ADR (23% vs. 17%, OR 1.49, 95%-CI 1.08–2.05, *p* = 0.01) and improved the detection of flat adenomas (OR 2.08, 95%-CI 1.35–3.20, *p* < 0.01) and SSA (OR 1.33, 95%-CI 1.01–1.74, *p* = 0.04) in a recent systematic review and meta-analysis [29]. Furthermore, the use of endocuff was associated with significant lower overall and proximal colon adenoma miss rates compared to conventional colonoscopy (14.7% vs. 38.4, and 10.4% vs. 38.9%) [30]. Another recent meta-analysis reported significantly lower adenoma and polyp miss rates in the proximal colon using add-on devices like cap, endocuff, endoring, third-eye-retroscope, and G-EYE, and a full-spectrum endoscopy system [31]. Procedures in our study were performed with inspection of the right-sided colon in simple forward view and without the use of any assistant devices.

Withdrawal time is defined by the time spent on withdrawal of the endoscope from the cecum to the anal canal and inspection of the entire bowel mucosa at negative colonoscopy without biopsy or therapeutic procedures [15]. A mean withdrawal time ≥ 6 min is associated with higher ADR and lower interval cancer rates and, therefore, represents an established quality indicator for screening colonoscopy [15]. It was also found to correlate with the detection of proximal SP [32]. The observation time in the proximal colon was a significant predictor for the detection of proximal adenomas and a minimum time span of at least 4 min was found to be sufficient for proximal adenoma detection [33]. Another prospective observational study reported a significant association between increased ADR and a withdrawal time of ≥2 min in the right-sided colon (OR 2.98, 95%-CI 1.72–5.15, *p* < 0.001) and ≥ 4 min in the proximal colon (OR 4.48, 95%-CI 3.15–6.36, *p* < 0.001) [34]. Interestingly, re-examination of the right-sided colon yielded a higher proximal ADR than a single examination with extended withdrawal time (33.1% vs. 23.6%, *p* = 0.045) in another study while total proximal withdrawal times were similar between both groups (4.29 ± 1.23 min vs. 4.34 ± 1.36 min, *p* = 0.74) [35].

In our study, endoscopists with high ADR had significantly higher IIR than endoscopists with low ADR which may reflect individual endoscopic skills to detect colonic lesions or a more vigilant examination of the colon. Unfortunately, data on withdrawal times, especially within the right-sided colon, were not available to incorporate this important variable in our analysis. Further limitations of our study also derive from its retrospective design. Quality of bowel preparation was assessed for the entire colon without separate assessment of bowel preparation in the proximal colon. Furthermore, photo or video documentation of cecal intubation was not assessed. Both variables may impact on detection rates of right-sided polyps and non-consideration may limit the findings of our study. On the other hand, our study comprises a large, well defined cohort of average risk individuals undergoing primary colonoscopy colorectal cancer screening, which was performed by experienced endoscopists.

## Conclusion

In our study, ileal intubation was not associated with higher detection rates for conventional adenomas and SP in the right-sided colon compared to cecal intubation. Therefore, advancing the colonoscope to the cecum without intubation of the terminal ileum may be sufficient for polyp detection in the right colon during screening colonoscopies.

## Data Availability

The datasets used and/or analysed during the current study are available from the corresponding author on reasonable request.

## Abbreviations

ADR: Adenoma detection rate
cAD: conventional adenomas
CI: Confidence interval
CIR: Cecal intubation rate
CRC: Colorectal cancer
DR: Detection rates
HP: Hyperplastic polyps
IIR: Ileal intubation rate
IQR: Interquartile range
OR: Odds ratio
SP: Serrated polyps
SSA: Sessile serrated adenoma
TSA: Traditional serrated adenoma

## References

[1] Bray Freddie, Ferlay Jacques, Soerjomataram Isabelle, Siegel Rebecca L., Torre Lindsey A., Jemal Ahmedin (2018). Global cancer statistics 2018: GLOBOCAN estimates of incidence and mortality worldwide for 36 cancers in 185 countries. CA: A Cancer Journal for Clinicians.

[2] Leggett B, Whitehall V (2010). Role of the serrated pathway in colorectal cancer pathogenesis. Gastroenterology.

[3] Snover DC, Ahnen D, Burt R, Bosman FT, Carneiro F, Hruban RH (2010). Serrated polyps of the colon and rectum and serrated (hyperplastic) polyposis. WHO classification of tumours of the digestive system.

[4] Schreuders EH, Ruco A, Rabeneck L (2015). Colorectal cancer screening: a global overview of existing programmes. Gut.

[5] Baxter NN, Goldwasser MA, Paszat LF (2009). Association of colonoscopy and death from colorectal cancer. Ann Intern Med.

[6] Brenner H, Hoffmeister M, Arndt V (2010). Protection from right- and left-sided colorectal neoplasms after colonoscopy: population-based study. J Natl Cancer Inst.

[7] Bressler B, Paszat LF, Chen Z (2007). Rates of new or missed colorectal cancers after colonoscopy and their risk factors: a population-based analysis. Gastroenterology.

[8] Lakoff J, Paszat LF, Saskin R (2008). Risk of developing proximal versus distal colorectal cancer after a negative colonoscopy: a population-based study. Clin Gastroenterol Hepatol.

[9] Singh H, Nugent Z, Demers AA (2010). The reduction in colorectal cancer mortality after colonoscopy varies by site of the cancer. Gastroenterology.

[10] Singh S, Singh PP, Murad MH (2014). Prevalence, risk factors, and outcomes of interval colorectal cancer: a systematic review and meta-analysis. Am J Gastroenterol.

[11] Lu FI, van Niekerk de W, Owen D (2010). Longitudinal outcome study of sessile serrated adenomas of the colorectum: an increased risk for subsequent right-sided colorectal carcinoma. Am J Surg Pathol.

[12] Robertson DJ, Greenberg ER, Beach M (2005). Colorectal cancer in patients under close colonoscopic surveillance. Gastroenterology.

[13] Sawhney MS, Farrar WD, Gudiseva S (2006). Microsatellite instability in interval colon cancers. Gastroenterology.

[14] Van Rijn JC, Reitsma JB, Stoker J (2006). Polyp miss rate determined by tandem colonoscopy: a systematic review. Am J Gastroenterol.

[15] Kaminski MF, Thomas-Gibson S, Bugajski M (2017). Performance measures for lower gastrointestinal endoscopy: a European Society of Gastrointestinal Endoscopy (ESGE) quality improvement initiative. Endoscopy.

[16] Lee TJW, Rees CJ, Blanks RG (2014). Colonoscopic factors associated with adenoma detection in a nation colorectal cancer screening program. Endoscopy.

[17] Jover R, Zapater P, Polania E (2013). Modifiable endoscopic factors that influence the adenoma detection rate in colorectal cancer screening colonoscopies. Gastrointest Endosc.

[18] Baxter NN, Sutradhar R, Forbes SS (2011). Analysis of administrative data finds endoscopist quality measures associated with postcolonoscopy colorectal cancer. Gastroenterology.

[19] Rupinska M, Wieszczy P, Franczyk R (2018). The effect of routine video-recording on colonoscopy quality indicators: a multicenter, cluster randomized controlled trial. Endoscopy.

[20] Thoufeeq MH, Rembacken BJ (2015). Meticulous cecal image documentation at colonoscopy is associated with improved polyp detection. Endosc Int Open.

[21] Schramm C, Mbaya N, Franklin J (2015). Patient- and procedure-related factors affecting proximal and distal detection rates for polyps and adenomas: results from 1603 screening colonoscopies. Int J Color Dis.

[22] Lee HS, Jeon SW (2015). Is retroflexion helpful in detecting adenomas in the right colon? A single center interim analysis. Intest Res.

[23] Chandran S, Parker F, Vaughan R (2015). Right-sided adenoma detection with retroflexion versus forward-view colonoscopy. Gastrointest Endosc.

[24] Kushnir VM, Oh YS, Hollander T (2015). Impact of retroflexion vs. second forward view examination of the right colon on adenoma detection: a comparison study. Am J Gastroenterol.

[25] Ai X, Qiao W, Han Z (2018). Results of a second examination of the right side of the colon in screening and surveillance colonoscopy: a systematic review and meta-analysis. Eur J Gastroenterol Hepatol.

[26] Kidambi Trilokesh D., Terdiman Jonathan P., El-Nachef Najwa, Singh Aparajita, Kattah Michael G., Lee Jeffrey K. (2019). Effect of I-scan Electronic Chromoendoscopy on Detection of Adenomas During Colonoscopy. Clinical Gastroenterology and Hepatology.

[27] Yoshida Naohisa, Inada Yutaka, Yasuda Ritsu, Murakami Takaaki, Hirose Ryohei, Inoue Ken, Dohi Osamu, Naito Yuji, Ogiso Kiyoshi, Morinaga Yukiko, Kishimoto Mitsuo, Konishi Eiichi, Itoh Yoshito (2018). Additional Thirty Seconds Observation with Linked Color Imaging Improves Detection of Missed Polyps in the Right-Sided Colon. Gastroenterology Research and Practice.

[28] Yoshida N, Inoue K, Yasuda R (2018). An additional 30-s observation of the right-sided colon with narrow band imaging decreases missed polyps: a pilot study. Dig Dis Sci.

[29] Desai M, Sanchez-Yague A, Choudhary A (2017). Impact of cap-assisted colonoscopy on detection of proximal colon adenomas: systematic review and meta-analysis. Gastrointest Endosc.

[30] Triantafyllou K, Polymeros D, Apostolopoulos P (2017). Endocuff-assisted colonoscopy is associated with a lower adenoma miss rate: a multicenter randomized tandem study. Endoscopy.

[31] Gkolfakis P, Tziatzios G, Facciorusso A (2018). Meta-analysis indicates that add-on devices and new endoscopes reduce colonoscopy adenoma miss rate. Eur J Gastroenterol Hepatol.

[32] Patel VD, Thompson WK, Lapin BR (2018). Screening colonoscopy withdrawal time threshold for adequate proximal serrated polyp detection rate. Dig Dis Sci.

[33] Klare P, Phlipsen H, Haller B (2017). Longer observation time increases adenoma detection in the proximal colon - a prospective study. Endosc Int Open.

[34] Jung Yunho, Joo Young-Eun, Kim Hyun Gun, Jeon Seong Ran, Cha Jae Myung, Yang Hyo-Joon, Kim Jong Wook, Lee Jun, Kim Kyeong Ok, Song Hye Kyung, Hwangbo Young, Shin Jeong Eun (2019). Relationship between the endoscopic withdrawal time and adenoma/polyp detection rate in individual colonic segments: a KASID multicenter study. Gastrointestinal Endoscopy.

[35] Guo CG, Zhang F, Ji R (2017). Efficacy of segmental re-examination of proximal colon for adenoma detection during colonoscopy: a randomized controlled trial. Endoscopy.

